# Programmed cell death ligand 1 measurement study in granulocyte colony-stimulating factor-producing lung cancer: an observational study

**DOI:** 10.1186/s12885-022-10065-w

**Published:** 2022-09-13

**Authors:** Kazuhito Miyazaki, Aya Shiba, Toshiki Ikeda, Yuko Higashi, Masaharu Aga, Yusuke Hamakawa, Yuri Taniguchi, Yuki Misumi, Yoko Agemi, Yukiko Nakamura, Tsuneo Shimokawa, Hiroaki Okamoto

**Affiliations:** Department of Respiratory Medicine, Yokohama Municipal Citizen’s Hospital, 1-1 Mitsuzawa Nishimachi, Kanagawa-ku, Yokohama, Kanagawa 221-0855 Japan

**Keywords:** G-CSF-producing lung cancer, PD-L1, Immune checkpoint inhibitors, Pembrolizumab, Immunohistochemistry

## Abstract

**Background:**

Granulocyte colony-stimulating factor (G-CSF)-producing lung cancer induces severe inflammation and a high white blood cell (WBC) count and is associated with poor prognosis. A recent case of G-CSF-producing lung adenocarcinoma showed high expression of programmed cell death ligand 1 (PD-L1) and was treated with pembrolizumab as first-line therapy, which was extremely effective. We hypothesized that G-CSF-producing lung cancers are associated with high PD-L1 expression.

**Methods:**

This retrospective study included patients diagnosed with lung cancer at Yokohama Municipal Citizen’s Hospital (Kanagawa, Japan) between 2009 and 2019. The PD-L1 status of 13 patients with high plasma G-CSF levels (≥40 pg/mL) was assessed by conducting immunohistochemical analysis of tissue samples.

**Results:**

Of the total patients, 11 were men and 2 were women, with a median age of 74 years (70–85 years). Four, five, and three patients had adenocarcinoma, squamous cell carcinoma, and others, respectively. The median G-CSF level and WBC count were 85.5 pg/mL (range, 40.8–484 pg/mL) and 15,550/μL (range, 6,190–56,800/μL), respectively. The PD-L1 tumor proportion scores (TPSs) were ≥50%, 1%–49%, and <1% in 9, 1, and 3 patients, respectively. The median overall survival time was 7.3 months. Pembrolizumab was administered in six patients as first-line treatment, with two patients showing partial response, one patient with stable disease, and three patients with progressive disease. All six patients had a PD-L1 TPS of ≥50%.

**Conclusion:**

G-CSF-producing lung cancers may be associated with increased PD-L1 expression. Although immune checkpoint inhibitors are an important treatment option for G-CSF-producing tumors, their effects are limited.

**Supplementary Information:**

The online version contains supplementary material available at 10.1186/s12885-022-10065-w.

## Introduction

Granulocyte colony-stimulating factor (G-CSF) is a cytokine that promotes granulocyte production and enhances neutrophil function [1, 2]. Recombinant human G-CSF is used for the treatment of neutropenia caused by chemotherapy and for increasing the number of donor peripheral blood stem cells. On the contrary, some rare types of solid tumors, including lung cancer, constantly produce G-CSF. G-CSF-producing tumors induce high fever, severe inflammation, and a high white blood cell (WBC) count. G-CSF-producing tumors are associated with poor prognosis, with a mean survival of several months [3, 4].

A recent case of G-CSF-producing lung adenocarcinoma showed high PD-L1 expression (TPS: 95%). The patient received pembrolizumab as first-line treatment, which was extremely effective.

In this study, we hypothesized that G-CSF-producing lung cancer is associated with high PD-L1 expression. Therefore, we retrospectively measured the PD-L1 status of patients with lung cancer and those with high plasma G-CSF levels (≥40 pg/mL).

## Materials and methods

### Patients and samples

A single-center, retrospective, observational study was performed at the Department of Respiratory Medicine, Yokohama Municipal Citizen’s Hospital (Kanagawa, Japan). Patients diagnosed with lung cancer at the hospital between 2009 and 2019 were included in this study. The PD-L1 status was determined by immunohistochemistry (IHC) using the PD-L1 IHC 22C3 PharmDx assay (Agilent Technologies, Santa Clara, California, United States). The tissue samples of patients with high plasma G-CSF levels (40 pg/mL) were used in the IHC. In two patients, serum interleukin-6 (IL-6) levels were measured simultaneously. The data of PD-L1 expression measured as a companion diagnostic marker were used (Supplementary Figure 1).

Informed consent was obtained from the patients through an opt-out method on the hospital’s website. None of the patients were excluded from the study. The data cut-off date was August 25, 2020.

### Cancer staging

Lung cancer staging was determined according to the 7^th^ edition of TNM for lung cancer or the 8^th^ edition of TNM for lung cancer, depending on the time of diagnosis.

### Statistical analysis

Overall survival (OS) was defined as the period from the date of diagnosis of lung cancer to the date of death from any cause. OS was determined using the Kaplan–Meier method.

### Institutional Review Board approval

The study was approved by the independent ethics committee of the Institutional Review Board (IRB) of Yokohama Municipal Citizens’ Hospital (no.19-03-05).

### Statement of human rights

All experiments involving human participants were performed in accordance with the ethical standards of the institutional and/or national research committee and the Declaration of Helsinki (1964) and its later amendments or comparable ethical standards.

## Results

### Patients’ characteristics

The patient’s baseline characteristics are shown in Table 1. Between January 2009 and December 2019, 2,681 patients were diagnosed with lung cancer in our hospital. Of them, 23 patients had already underwent measurement of serum G-CSF expression, as they were suspected of having G-CSF-producing lung cancer due to the presence of fever, increased WBC count, high inflammatory response, and diffuse accumulation of 18F-fluorodeoxyglucose (FDG) in the bone marrow as shown on positron emission tomography/computed tomography (PET/CT) scan. Of the 23 patients, 4 had low G-CSF levels and 19 had high G-CSF levels. Of the 19 patients, 1 received G-CSF (Filgrastim) therapy, 4 had no tissue samples, and 1 was excluded as PD-L1 staining could not be performed owing to the small amount of residual tissue. Ultimately, 13 patients were enrolled in the study (Supplementary Figure 1). With regard to the smoking history, 7 patients were current smokers, while 6 were ex-smokers. The median blood G-CSF level was 85.5 pg/mL (range: 40.8–484 pg/mL), while the median WBC count was 15,550/μL (range: 6,190–56,800/μL). The clinical stage at diagnosis was determined; six patients had stage III cancer (stage IIIA or IIIB), while seven had stage IV cancer (stage IV of the 7th edition and IVA and IVB of the 8th edition of TMN). The Eastern Cooperative Oncology Group Performance Status (ECOG-PS) scores at diagnosis were 1 in six patients, 2 in six patients, and 3 in one patient. The serum IL-6 level was measured in 2 of the 13 patients, and the levels were high (61.9 pg/mL and 73.8 pg/mL, respectively).

**Table 1:** Baseline characteristics of patients

age, yr (Range)	74 (70-85)	
sex		
male	11	84.6
female	2	15.4
smoking status		
current smoker	7	53.8
ex-smokers	6	46.2
Median G-CSF level		
pg/mL (Range)	85.5 (40.8-484)	
Median WBC count		
/μL (Range)	15,550 (6,190-56,800)	
Clinical stage at diagnosis		
StageIII	6	46.2
StageIV	7	53.8
ECOG-PS at diagnosis		
1	6	46.2
2	6	46.2
3	1	7.7

*G-CSF* Granulocyte colony-stimulating factor, *WBC* white blood cell, *ECOG-PS* Eastern Cooperative Oncology Group Performance Status

### Pathological diagnosis

The following pathological subtypes were diagnosed: adenocarcinoma (4); squamous cell carcinoma (Sq) (5); poorly differentiated carcinoma (2); non-small cell lung cancer, not otherwise specified (1); and sq+ small cell lung cancer (SCLC) (1) (Table 2). Seven of the 13 patients underwent a driver mutation test, EGFR PCR was performed in seven patients, ALK-IHC was performed in seven patients, and ROS1 RT-PCR was performed in two patients, all of which showed negative results.

**Table 2:** Pathological diagnosis

	N	(%)
Adenocarcinoma	4	30.8
Squamous cell carcinoma (Sq)	5	38.5
Poorly differentiated carcinoma	2	15.4
NOS	1	7.7
Sq + Small cell lung cancer	1	7.7

*NOS* non-small cell lung cancer, not otherwise specified

### PD-L1 IHC

The PD-L1 status was determined using IHC. The PD-L1 TPSs were ≥50%, 1%–49%, and <1% in 9 (69.2%), 1 (7.7%), and 3 (23.1%) patients, respectively (Table 3).

**Table 3:** Programmed cell death ligand 1 (PD-L1) immunohistochemistry

PD-L1 IHC (TPS)	N	(%)
50≥	9	69.2
1-49	1	7.7
1<	3	23.1

*IHC* immunohistochemistry, *TPS* tumor proportion score

### Therapeutic effect of immune checkpoint inhibitors

During the study period, 8 of the 13 patients received immune checkpoint inhibitors (ICIs), 6 patients received pembrolizumab, and 2 patients received nivolumab (Table 4). The PD-L1 TPS was 50% or higher in all patients who received pembrolizumab as first-line treatment. As of August 25, 2020, three patients continued to receive treatment. Among patients treated with pembrolizumab, two patients achieved partial response (PR), one patient had stable disease (SD), and three patients had progressive disease (PD). Two of the three PD patients had high IL-6 levels. The response rate of patients who received pembrolizumab as first-line treatment was 33.3%, and the disease control rate was 50%. Two patients who achieved PR were still receiving pembrolizumab at the time of data cutoff and survived for 796 and 329 days, respectively.

**Table 4:** Therapeutic effect of immune checkpoint inhibitors

Patient characteristics		
N=8	N	(%)
1st line	6	
Pembrolizumab	6	100
2nd line	2	
Nivolumab	2	100
Pembrolizumab		
PR	2	33.3
SD	1	16.7
PD	3	50
Nivolumab		
PD	2	100

*PR* partial response, *SD* stable disease, *PD* progressive disease

All patients treated with nivolumab had PD; one patient had a PD-L1 TPS of 30%, another patient had Sq and SCLC, and both Sq and SCLC had a PD-L1 TPS of <1%.

### Median OS

**Fig. 1:**
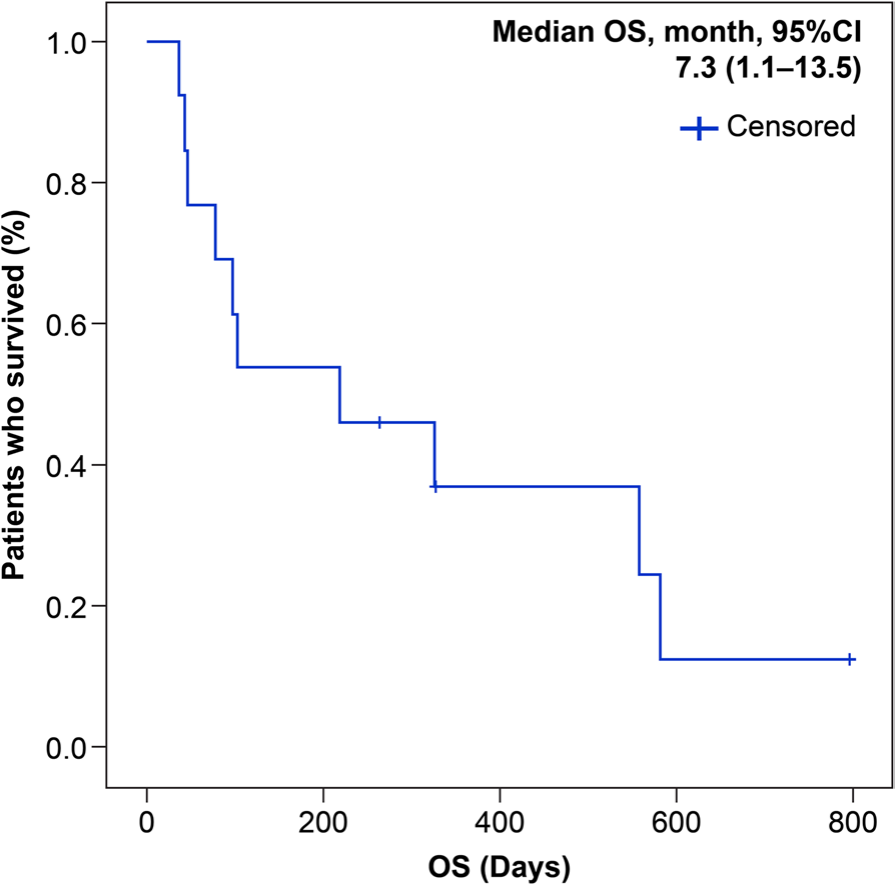
Kaplan–Meier curves of overall survival (OS). The median OS of patients with granulocyte colony stimulating factor-producing lung cancer in our hospital (*N*=13) was 7.3 months (95%CI: 1.1–13.5)

The median OS from the time of diagnosis was estimated using the Kaplan–Meier method. On August 25, 2020, the median OS was 219 days (7.3 months) (Fig. 1).

### PET/CT

All 13 patients underwent PET/CT at the time of diagnosis, which showed diffuse accumulation of FDG in the bone marrow in 12 of the 13 patients (Supplementary Figure 2).

## Discussion

To the best of our knowledge, this study was the first to confirm the relationship between G-CSF-producing lung cancer and PD-L1 expression. G-CSF-producing lung cancer is associated with high fever, increased inflammatory response, and increased WBC count. It progresses rapidly and is associated with an extremely poor prognosis.

The diagnostic criteria for G-CSF-producing malignant tumor are as follows: (1) marked increase in WBC count without infection or other diseases, (2) increased serum G-CSF levels, (3) reduction in WBC count after tumor resection, and (4) G-CSF-positive staining of tumor tissues on IHC [5]. On the contrary, as immunostaining for G-CSF was not available in our hospital, this study was only performed in patients with increased serum G-CSF levels due to G-CSF-producing lung cancer. Between 2009 and 2019, 13 patients had increased serum levels of G-CSF that could be immunostained for PD-L1. As shown in Table 3, the PD-L1 TPSs were ≥50%, 1%–49%, and <1% in 9 (69.2%), 1 (7.7%), and 3 (23.1%) patients, respectively. This result is clearly higher than the percentage of patients in previous studies with a PD-L1 TPS of ≥50% (30%) [6, 7]. Although this study included a small number of participants, PD-L1 expression is highly likely upregulated in those with G-CSF-producing lung cancers.

Several studies reporting the abnormal accumulation of G-CSF-producing tumors in the bone marrow examined on FDG-PET are available, which is considered useful for diagnosis [8–11]. In the present study, 12 of 13 patients with G-CSF-producing lung cancers showed diffuse accumulation of FDG in the bone marrow, and all 4 patients with a confirmed diagnosis of G-CSF-producing lung cancer showed FDG accumulation. As previously reported, FDG accumulation in the bone marrow is useful in differentiating patients with this disease.

Treatment of advanced-stage lung cancer has dramatically changed since the discovery of the EGFR gene mutation in the 2000s and various driver gene mutations [12–14]. Furthermore, nivolumab, a programmed death-1 antibody, was approved for use in December 2015 in Japan; multiple ICIs, including pembrolizumab, have also been approved [6, 15–17]. As regards the therapeutic effect of ICIs, pembrolizumab used as first-line treatment had a response rate of 33.3% and a disease control rate of 50%, whereas two patients using nivolumab as second-line treatment had PD. The PD-L1 TPS was ≥50% in all patients who received pembrolizumab as first-line treatment but was lower than the response rate reported in the KEYNOTE-024 trial. Although this finding is ambiguous owing to the small number of cases, the underlying mechanisms involved in the occurrence of resistance to some ICIs may contribute to the development of G-CSF-producing lung cancer.

This study showed that ICIs are a useful treatment option for G-CSF-producing lung cancer, although their efficacy is limited. In patients with G-CSF-producing lung cancers, the ECOG-PS often decreases due to high fever and increased inflammation. Hence, the disease must be diagnosed as early as possible and chemotherapy plus ICI or ICI combinations should be used as treatment instead of ICI alone to achieve greater efficacy. Therefore, early diagnosis is important for maintaining good ECOG-PS. The current diagnostic criteria for G-CSF-producing tumors are not useful for selecting the appropriate treatment for rapidly progressing G-CSF-producing lung cancer. This is because many institutions, including our hospital, use G-CSF levels and results of immunostaining to assess for G-CSF as criteria, which do not provide immediate results. In the case that led to this study, the WBC count was normal at the time of diagnosis; however, fever, increased inflammatory response, PET-CT findings, and high G-CSF count led to the suspicion of G-CSF-producing lung cancer. In this case, leukocytes increased to 10,000 at the start of treatment; in response to pembrolizumab treatment, the serum G-CSF level, which was 180 pg/mL prior to the start of treatment, decreased below the measured sensitivity (19.5 pg/mL) after the initiation of treatment. In other words, depending on the progression status, patients with G-CSF-producing lung cancers may not necessarily have high WBC levels. However, as mentioned earlier, PET-CT findings are useful for differentiating G-CSF-producing lung cancers. Therefore, we hypothesized that the presence of diffuse bone marrow accumulation on PET-CT, high WBC count, increased inflammatory response (increased C-reactive protein), and fever should be considered for diagnosing G-CSF-producing tumors.

This study has some limitations. First, it was a small, uncontrolled, single-center study with no comparison groups. Second, the G-CSF level was measured at the discretion of the attending physician, and not all patients with suspected G-CSF-producing lung cancer were tested. Third, immunostaining for G-CSF was not performed; hence, it was possible that the reliable G-CSF-producing tumors were not collected. The reason for this is that the conditions for immunostaining of G-CSF were not available at our hospital owing to the lack of insurance coverage. Fourth, this study enrolled patients who were diagnosed with this disease in 2009–2019, and data of the oldest specimen from the 13 patients were finally extracted in 2012; those from 2012 had a PD-L1 TPS of 95%, while all seven patients from 2018 and later had a TPS of 50% or higher. However, only two of the six specimens from 2017 and earlier had a TPS of 50% or higher. This finding suggests that the older specimens may have had inferior staining and may have had lower PD-L1 expression than they actually did. Finally, it remained unclear whether the production of G-CSF by the tumor reduced the effect of ICI. IL-6 levels may be increased in G-CSF-producing tumors [11]. The IL-6 levels were measured in two patients in our hospital, and both patients showed increased levels. Neither of these two patients showed positive response to pembrolizumab treatment. In addition, IL-6 is one of the causes of ICI resistance [18]. The co-expression of G-CSF and IL-6 possibly contributed to the occurrence of ICI resistance.

## Conclusions

G-CSF-producing lung cancers may be associated with increased PD-L1 expression. Although ICIs are important treatment alternatives for G-CSF-producing lung cancer, their efficacy is limited. Hence, a large-scale study is warranted to evaluate the effects of ICI + chemotherapy on G-CSF-producing lung cancer.

## Supplementary Information

**Additional file 1.** 

## Data Availability

The datasets generated and/or analyzed during the current study are not publicly available because they include some indirect identifying information (age, sex, ECOG PS, TNM classification, driver mutation status, date of cancer diagnosis, initial date of medication, date of disease progression, and date of death) but are available from the corresponding author upon reasonable request.

## Abbreviations

G-CSF: Granulocyte colony stimulating factor
PD-L1: Programmed cell death ligand 1
TPS: Tumor proportion score
WBC: White blood cell
IHC: Immunohistochemistry
OS: Overall survival
IRB: Institutional Review Board
FDG: 18F-fluorodeoxyglucose
PET/CT: Positron emission tomography/computed tomography
ECOG-PS: Eastern Cooperative Oncology Group Performance Status
Sq: Squamous cell carcinoma
SCLC: Small cell lung cancer
ICIs: Immune checkpoint inhibitors
PR: Partial response
SD: Stable disease
PD: Progressive disease

## References

[1] Metcalf D (1985). The granulocyte-macrophage colony-stimulating factors. Science..

[2] Nagata S, Tsuchiya M, Asano S (1986). Molecular cloning and expression of cDNA for human granulocyte colony-stimulating factor. Nature..

[3] Asano S, Urabe A, Okabe T (1977). Demonstration of granulopoietic factor(s) in the plasma of nude mice transplanted with a human lung cancer and in the tumor tissue. Blood..

[4] Morita Y, Sakaguchi T, Ida S (2020). Aggressive Recurrent Pancreatic Cancer Producing Granulocyte Colony-Stimulating Factor. Case Rep Gastroenterol..

[5] Joshita S, Nakazawa K, Sugiyama Y (2009). Granulocyte-Colony Stimulating Factor-Producing Pancreatic Adenosquamous Carcinoma Showing Aggressive Clinical Course. Intern Med..

[6] Reck M, Rodríguez-Abreu D (2016). Pembrolizumab versus Chemotherapy for PD-L1-Positive Non-Small-Cell Lung Cancer. N Engl J Med..

[7] Garon EB, Rizvi NA, Hui R (2015). Pembrolizumab for the treatment of non-small-cell lung cancer. N Engl J Med..

[8] Morooka M, Kubota K, Murata Y (2008). (18)F-FDG-PET/CT findings of granulocyte colony stimulating factor (G-CSF)-producing lung tumors. Ann Nucl Med..

[9] Kuroshima T, Wada M, Sato T (2018). G-CSF producing oral carcinoma with diffuse uptake of FDG in the bone marrow: A case report. Oncol Lett..

[10] Makino T, Hata Y, Otsuka H (2017). Diffuse fluorodeoxyglucose-positron uptake in the bone marrow of a patient with granulocyte colony-stimulating factor-producing pleomorphic carcinoma of the lung: A case report. Mol Clin Oncol..

[11] Yoshida Y, Sibusa T, Ishii Y (2019). Granulocyte Colony-stimulating Factorf and Interleukin-6-producing Large-cell Carcinoma of the Lung with Sarcomatoid Changes Suggestive of Epithelial-mesenchymal Transition: An Autopsy Case Report. Intern Med..

[12] Mitsudomi T, Morita S, Yatabe Y (2010). Gefitinib versus cisplatin plus docetaxel in patients with non-small-cell lung cancer harbouring mutations of the epidermal growth factor receptor(WJTOG3405): an open label, randomised phase 3 trial. Lancet Oncol..

[13] Solomon BJ, Mok T, Kim DW (2014). First-line crizotinib versus chemotherapy in ALK-positive lung cancer. N Engl J Med..

[14] Hida T, Nokihara H, Kondo M (2017). Alectinib versus crizotinib in patients with ALK-positive non-small-cell lung cancer(J-ALEX): an open-label, randomised phase 3 trial. Lancet..

[15] Mok TSK, Wu YL, Kudaba I (2019). Pembrolizumab versus chemotherapy for previously untreated, PD-L1-expressing, locally advanced or metastatic non-small-cell lung cancer (KEYNOTE-042): a randomised, open-label, controlled, phase 3 trial. Lancet..

[16] Brahmer J, Reckamp KL, Baas P (2015). Nivolumab versus docetaxel in Advanced squamous-Cell Non-Small-Cell Lung Cancer. N Engl J Med..

[17] Borghaei H, Paz-Ares L, Horn L (2015). Nivolumab versus docetaxel in Advanced nonsquamous Non-Small-Cell Lung Cancer. N Engl J Med..

[18] Keegan A, Ricciuti B, Garden P (2020). Plasma IL-6 changes correlate to PD-1 inhibitor responses in NSCLC. J Immunother Cancer..

