# Supplementary material for: Programmed cell death ligand 1 measurement study in granulocyte colony-stimulating factor-producing lung cancer: an observational study
Source: BMC Cancer. 2022 Sep 13;22:977. doi: 10.1186/s12885-022-10065-w (PMC9469597; doi:10.1186/s12885-022-10065-w)
Supplement: Supplementary file 1 — Additional file 1. [file 12885_2022_10065_MOESM1_ESM.docx]

Supplementary Figure 1. Diagram of patient selection.

A diagram of patient selection in this study is shown.


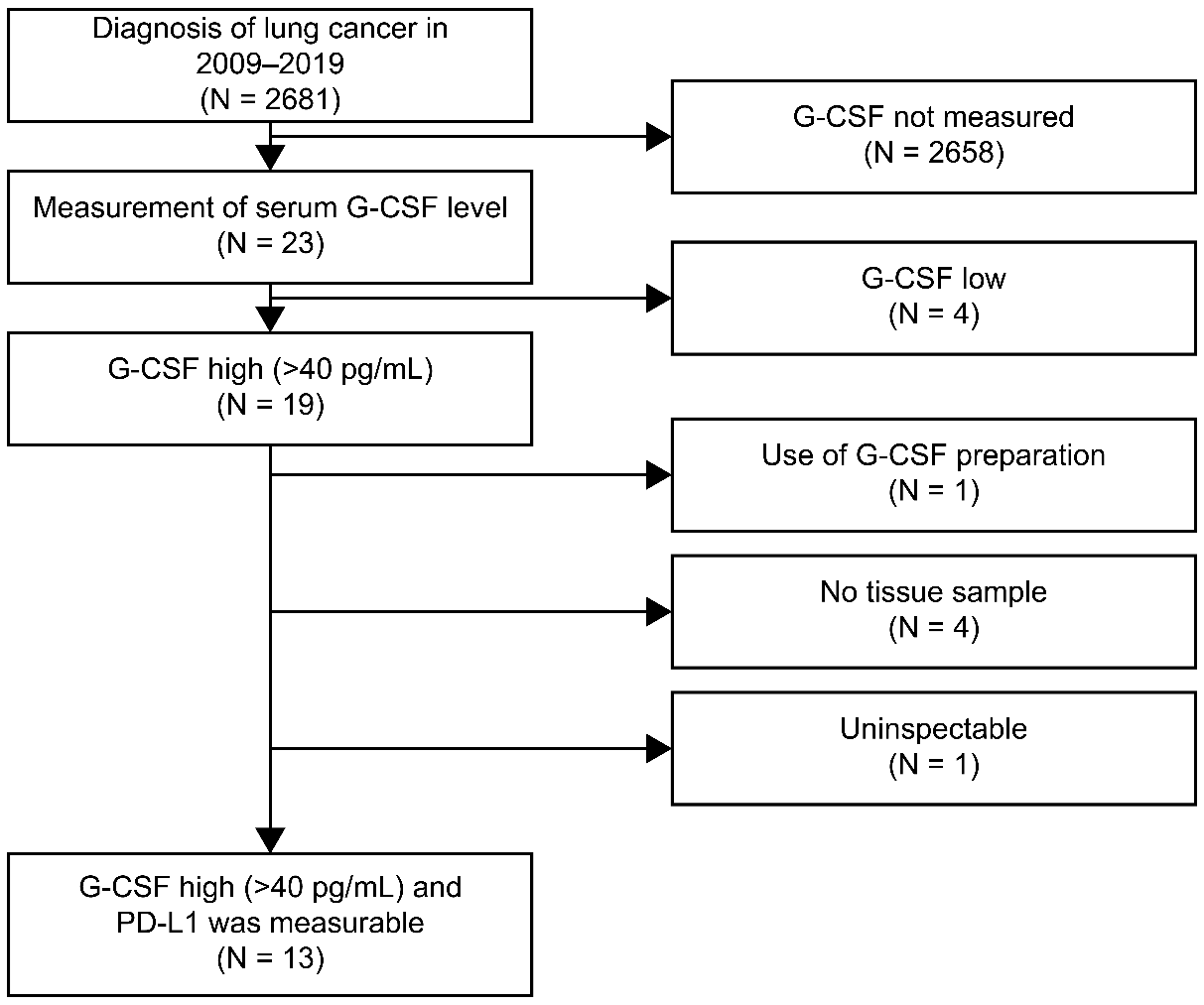


Supplementary Figure 2. PET/CT finding of the confirmed granulocyte colony stimulating factor-producing lung cancer patient.

The patient was a 70-year-old woman. In addition to the primary lesion in the right lower lobe, diffuse FDG accumulation was observed in the spine and pelvic bones.


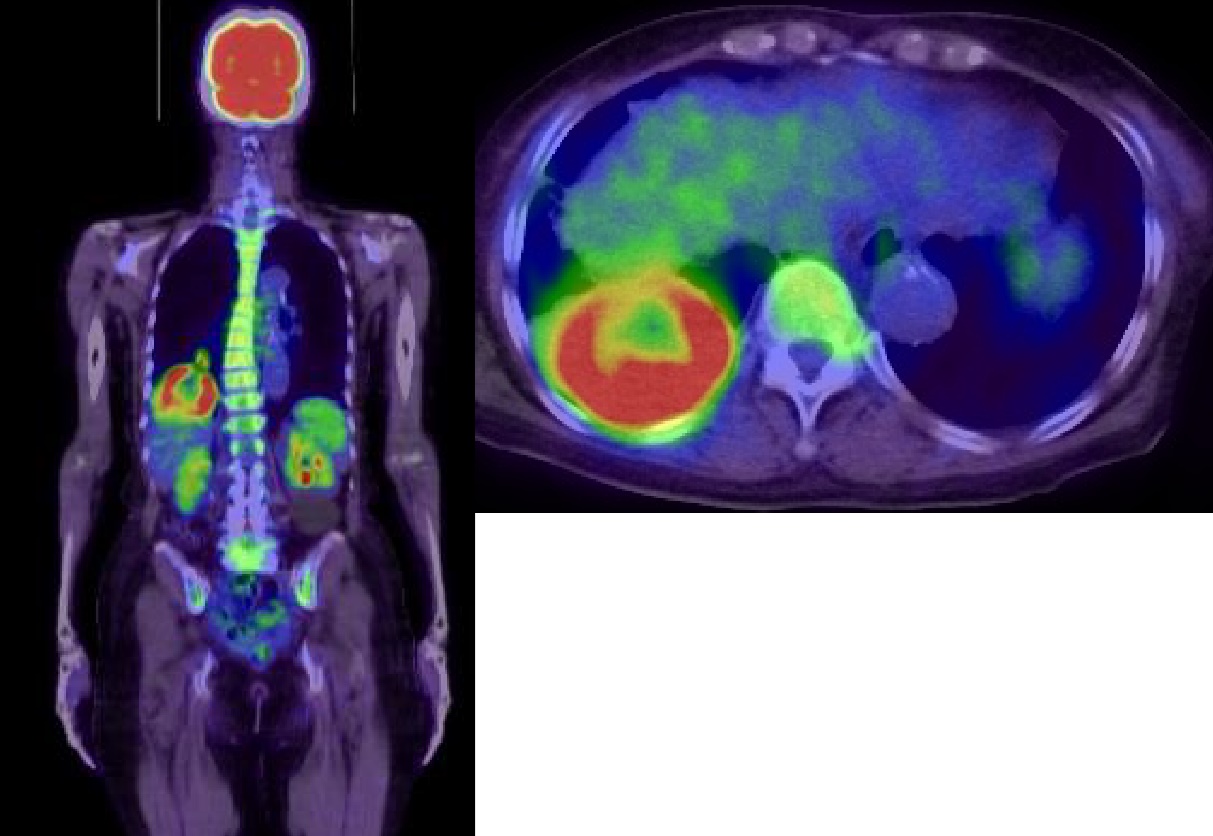


PET/CT: Positron emission tomography/computed tomography, FDG: 18F-fluorodeoxyglucose
